# *Impatiensjenjittikuliae* (Balsaminaceae), a new species from Thailand

**DOI:** 10.3897/phytokeys.124.33607

**Published:** 2019-06-21

**Authors:** Saroj Ruchisansakun, Piyakaset Suksathan

**Affiliations:** 1 Department of Plant Science, Faculty of Science, Mahidol University, Bangkok, Thailand Department of Plant Science, Faculty of Science, Mahidol University Bangkok Thailand; 2 Queen Sirikit Botanic Garden, The Botanical Garden Organization, Chiang Mai, Thailand Queen Sirikit Botanic Garden Chiang Mai Thailand

**Keywords:** *
Uniflorae
*, lithophytic, endemic, critically endangered

## Abstract

*Impatiensjenjittikuliae* Ruchis. & Suksathan, a new species from a limestone area in Thasongyang District, Tak Province, Northern Thailand, is described and illustrated. This endemic new species is distinguished from the most similar, *I.lacei* Hook.f. through having pilose lateral sepals vs glabrous, and by the absence of long hairs along the lamina margin. Its pollen and seed morphology, stem anatomy, and pollination ecology are also observed. Furthermore, its conservation status as Critically Endangered is also assessed.

## Introduction

Balsaminaceae consists of *Hydrocera* Blume ex Wight & Arnott (1834: 140) with only a single species, *H.triflora* (L.) Wight & Arnott (1834: 140), and *Impatiens* L. (1753: 937), comprising over 1,000 species (Ruchisansakun et al. 2018). In Southeast Asia, many new *Impatiens* species were recently described (Souvannakhoummane and Suksathan 2015; Ruchisansakun et al. 2017; Ruchisansakun et al. 2018; Suksathan and Triboun 2009).

In Thailand, sixty-one native *Impatiens* species have been enumerated in previous works (Shimizu 1970, 1977, 1991, 2000; Shimizu and Suksathan 2004; Suksathan and Triboun 2009; Ruchisansakun et al. 2014). In 2017, the first author cited here noticed an unnamed *Impatiens* from Tak Province via Weerayuth Laohajinda’s Facebook and later on traveled to examine the plant in 2018. After a detailed study, it turned out to be a species new to science. It is therefore described here.

## Material and methods

Fieldwork was conducted in October 2018. The plants were prepared for making herbarium specimens. Each part of the flower was separately glued on the hard paper and dried separately. All parts were measured and described in line with terminology in Ruchisansakun et al. (2018). For a palynological study, mature pollen grains were collected, air-dried, and fixed to aluminum stubs, then sputter-coated with gold. Micrographs were taken with a Field Emission Scanning Electron Microscope (FE-SEM) (Hitachi SU8010). The pollen grains and seeds were measured by ImageJ and described according to the terminology of pollen grains and seeds (Janssens et al. 2012). For an anatomical study, the fresh stem was dissected at the base and stained by diluted Safranin-O for 20 sec, and observed under a light microscope.

## Result

### Taxonomy

#### Impatiens jenjittikuliae

Taxon classificationPlantaeORDOFAMILIA

Ruchis. & Suksathan sp. nov.

urn:lsid:ipni.org:names:77198714-1

**Figure 1.**
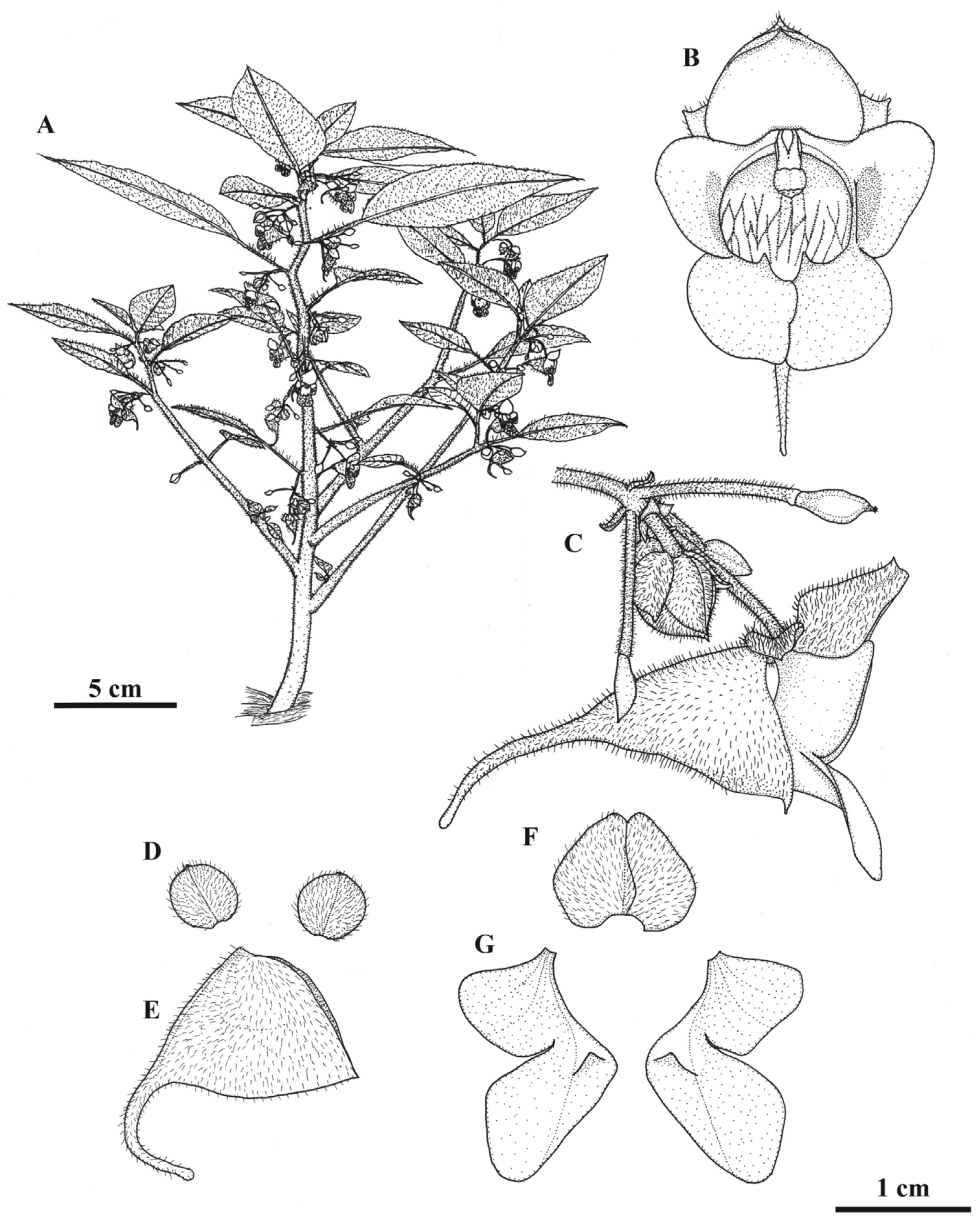
*Impatiensjenjittikuliae*. **A** Habit **B** Flower, front view **C** Inflorescence with flower in lateral view **D** Lateral sepals **E** Lower sepal **F** Dorsal petal **G** Lateral united petals. Drawn by Saroj Ruchisansakun.

**Figure 2.**
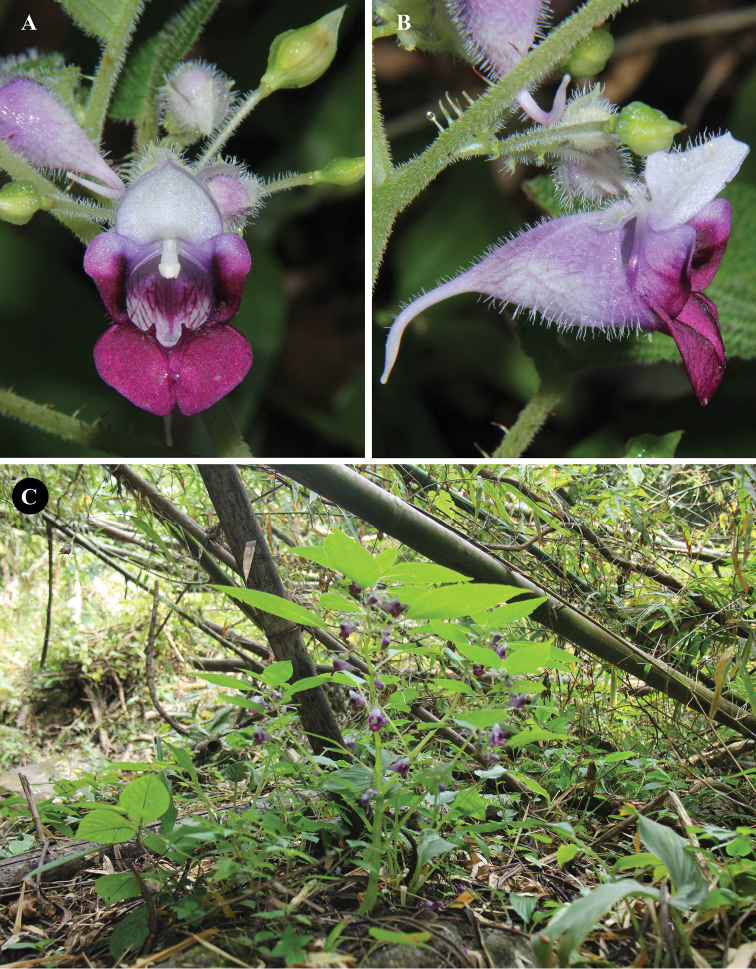
*Impatiensjenjittikuliae***A** flower, front view **B** flower, lateral view **C** habit *in situ*. Photographs by Saroj Ruchisansakun.

**Figure 3.**
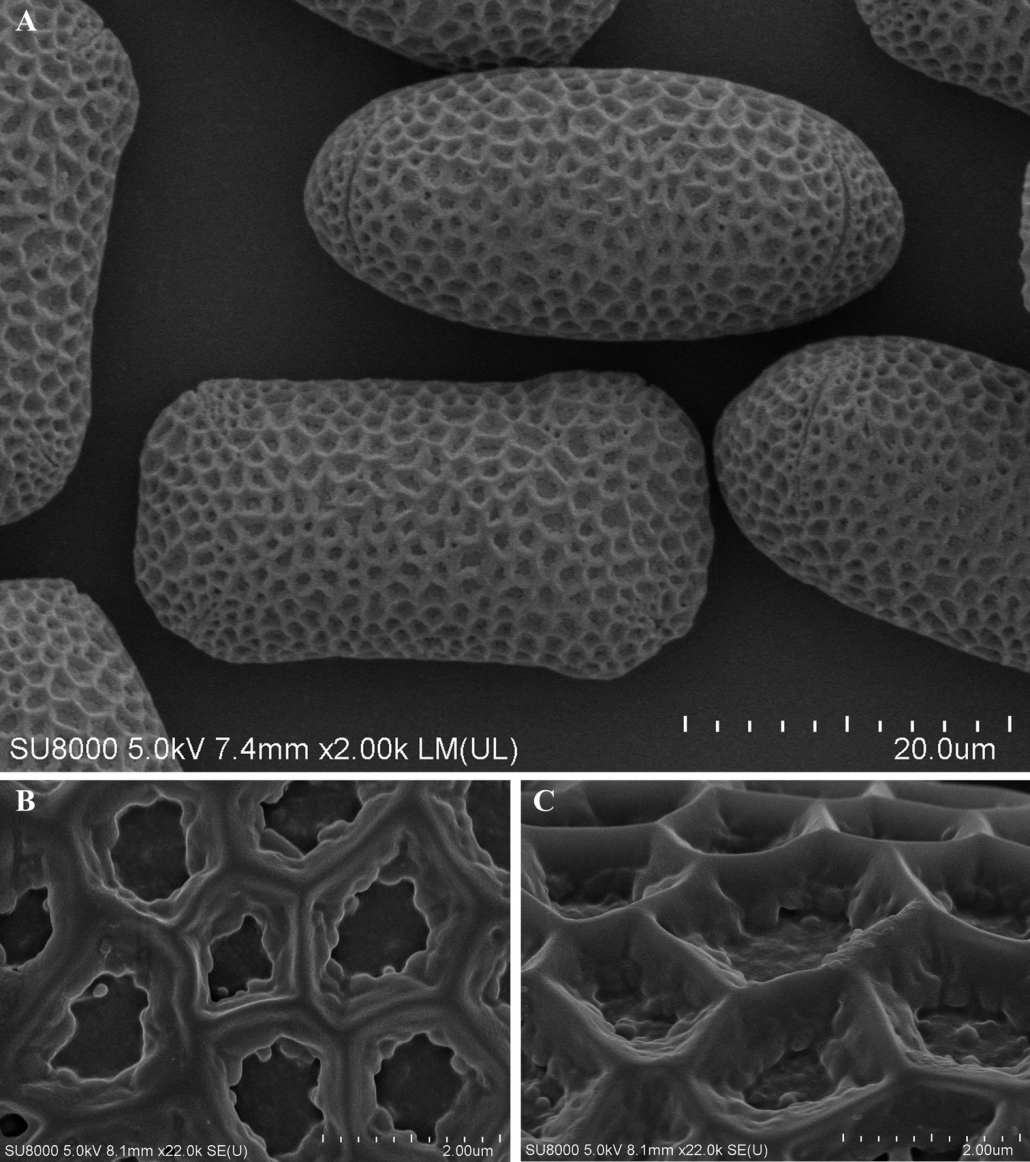
Pollen of *Impatiensjenjittikuliae* (FE SEM) **A** entire pollens **B, C** sexine ornamentation.

**Figure 4.**
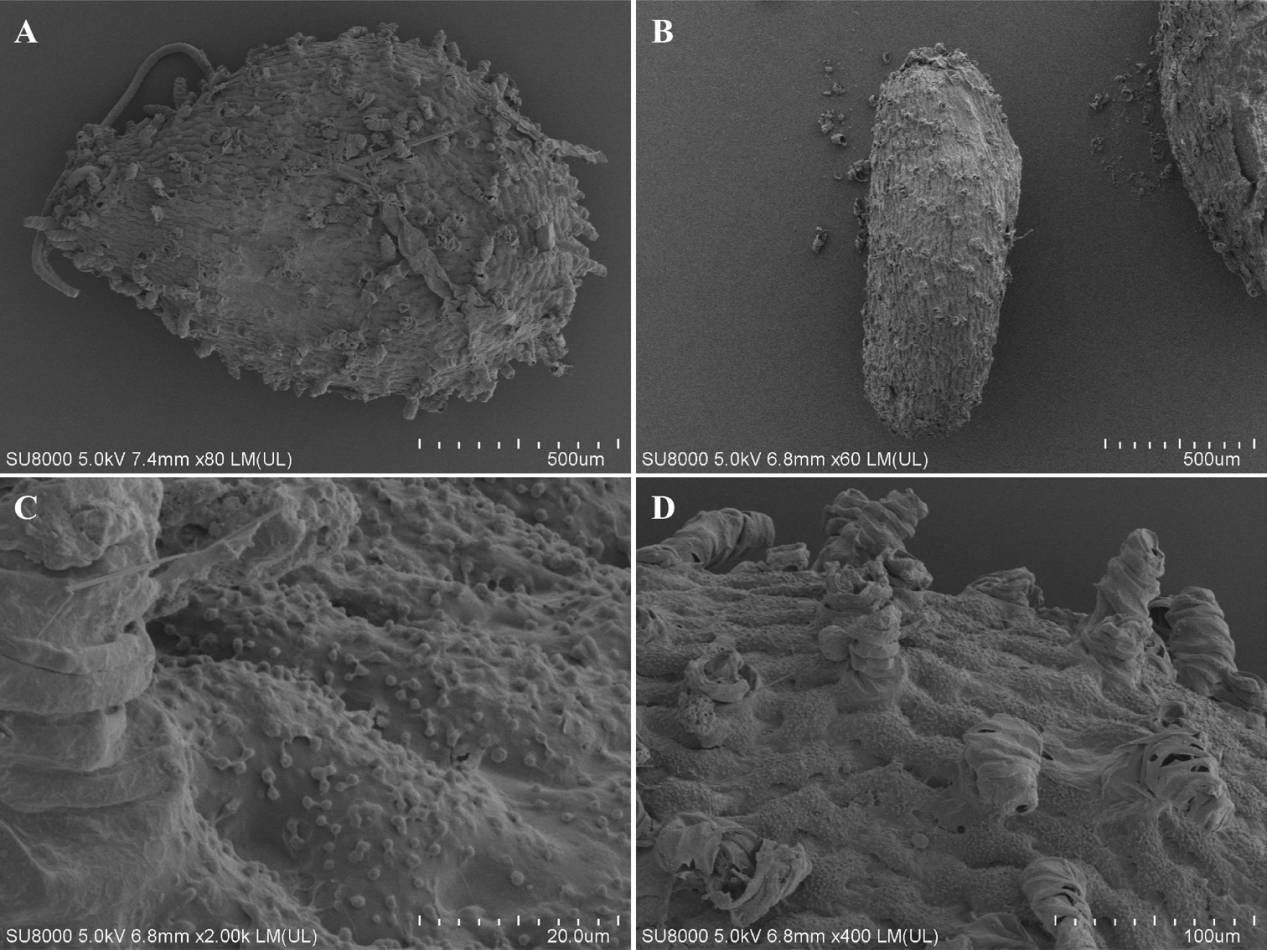
Seed of *Impatiensjenjittikuliae* (FE SEM). **A, B** Entire seeds **C** Inflated cells with granulate walls **D** Thick finger-like cells.

**Figure 5.**
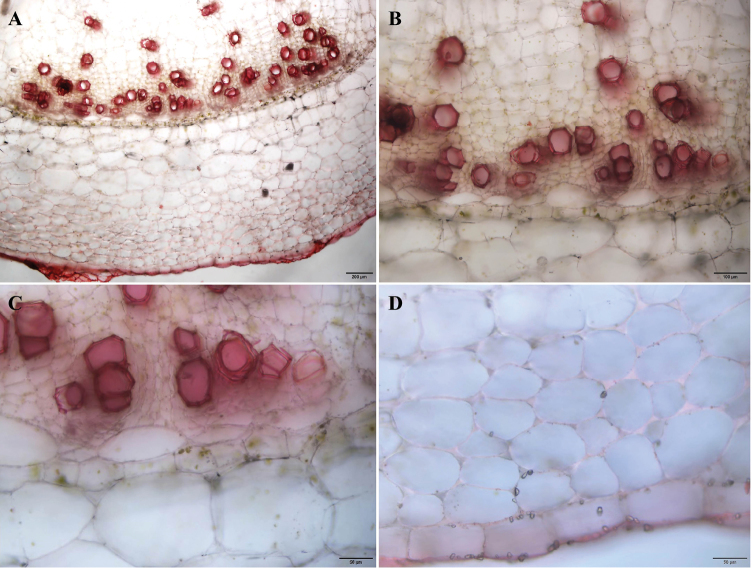
Stem of *Impatiensjenjittikuliae*, transverse sections.

Figs 1 , 2 , 3 , 4 , 5

##### Diagnosis.

*Impatiensjenjittikuliae* is most similar to *I.lacei* Hook.f. It differs from *I.lacei* by its densely pilose lateral sepal (versus glabrous) and by having no long hairs along its lamina margin (versus distinct long hairs especially along the lower-half of leaf margin).

##### Type.

THAILAND. Tak Province, Thasongyang District [17°30'1"N, 98°3'60"E], limestone area near waterfall in mixed deciduous forest at 540 m alt., 20 October 2018, *S. Ruchisansakun* 900 (holotype: QBG; isotypes: BK, BKF, Mahidol University Herbarium)

##### Description.

Lithophytic, annual herb, up to 6–30 cm tall. Stem erect, up to 1.2 cm in diam., cylindrical, branched, green, densely pilose with short white hairs. Leaves spirally arranged. Petiole 3–7.5 cm long, ca. 2.5 mm in diam., pale green to green to pink, pilose; with 5–7 pairs of long hairs on petiole, up to 2 mm long, green, sometime with red tips. Lamina 9–20 × 3–7 cm, ovate to elliptic, apex acute, base cuneate, margin shallowly serrate, adaxial green, abaxial pale green, pilose on both sides; lateral veins 10–12 pairs. Inflorescence raceme, axillary, 8–12 florets; peduncle 7–10 mm long, 1.5–2 mm in diam., pale green, densely pilose; rachis 8–20 mm long, ca. 1.5 mm in diam., pale green, densely pilose, hairs shorter than those on peduncle. Flowers ca. 20 × ca. 15 mm, ca 3 mm deep, pinkish white with reddish purple lip. Bracts ca. 1 × 0.5 mm, linear to narrowly lanceolate, apex acute, base cuneate, green, caducous, abaxial densely pilose with white hairs. Pedicel 12–15 mm long, less than 1 mm in diam., pink, densely pilose with white hairs. Lateral sepals 2, 5–6 × 6–7 mm, free, broadly ovate, the apex mucronate, the base truncate, pale pink, abaxially densely pilose with white hairs. Lower sepal 11–13 × 8–10 mm, ca. 14–17 mm deep, broadly navicular to subsaccate, apex acuminate and mucronate, white with green tip, densely pilose to strigose outside with long white hairs, distal part gradually constricted into a curved spur, 14–15 mm long, white to pale pink. Dorsal petal 11–12 × 14–15 mm, broadly ovate to obcordate, cucullate, apex emarginate and mucronate, base cordate, white to pale pink, densely pilose with white hairs, abaxial midvein with a white crescent-shaped crest, 1–1.5 mm high. Lateral united petals 20–24 mm long, free: the upper petals 9–10 × 10–11 mm, broadly oblong, apex truncate, base cuneate, upper outer part white to pale pink, lower inner part dark purple; the lower petals 17–19 × 8–10 mm, oblong, apex truncate, pink to reddish-purple; with a pink auricle at the base, ca 1 mm high. Stamens 5; filaments 4–5 mm long, white; anthers white. Ovary ca 4 mm long, 1.5–2 mm in diam., short fusiform, 5-carpellate, green, glabrous. Fruits, short fusiform capsule, 11–12 mm long, 6–8 mm in diam., subglobose, 5–lobed, green, glabrous. Seeds ca. 20 per fruit, ca. 1.34 mm long, ovoid, brown.

***Pollen morphology***: Pollen grains 4-colpate (Fig. 3A). Equatorial view oblong, ca 35 × 18–19 μm (length/width = 1.89); Polar view nearly elliptic, ca. 17 μm thick, colpi four, linear, ca 9–10 μm (Fig. 3A); surface entirely covered with numerous irregular lumens, 1.2–2 μm diam, lumens deep, sparsely granulate (Fig. 3B); muri slightly straight, joint of muri slightly corniculate (Fig. 3C).

***Seed morphology***: Brown ovoid, ca 1.34 × 0.93 mm, ca 0.51 μm thick (length/width = 1.44) (Fig. 4). Seed coat a composite of two types, thick finger-like cells, and inflated cells with granulate walls (Figs 4C, D).

***Stem anatomy***: Stem herbaceous, without lignification (Figs 5A–C). Only angular collenchyma for stem-strengthen were found (Fig. 5D).

##### Phenology.

Flowering from Oct. to Nov.; fruiting Oct. from Nov.

##### Distribution.

The new species is only known from the type locality in Tak Province, Thailand.

##### Ecology.

*Impatiensjenjittikuliae* grows on limestone close to waterfall in a mixed deciduous forest, 520–600 m elevation (pers. obs.).

##### Proposed IUCN conservation assessment.

Critically Endangered B1ab (i, ii, iii) + 2ab (i, ii, iii). This species is only known from the type locality; the extent of occurrence is estimated to be less than 5 km, where it occurs as a small population (IUCN 2012).

##### Etymology.

The new species is named in honor of Dr. Thaya Jenjittikul who encouraged the first author to step in and study this lovely plant family.

##### Pollination ecology.

**Figure 6.**
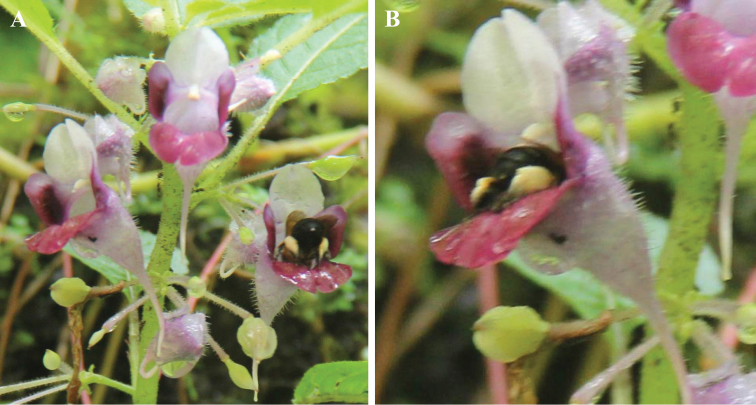
Floral visitation by bee in the locality of *Impatiensjenjittikuliae*.

The author observed five visitations by bees from the family Apidae (identified by an entomologist, Pornpimon Tangtorwongsakul) during the expeditions. The size of bee body fit well with the floral entrance (Fig. 6). Moreover, the floral structure of this new species is similar to other bee-pollinated species, e.g. *I.psittacina* (Ruchisansakun et al. 2016). Hence, we concluded that it is a bee-pollinated species.

## Discussion

*Impatiensjenjittikuliae* is similar to *I.lacei* and the other species closely related to *I.pulchra* Hook.f (= *I.mengtszeana* Hook.f. in Ruchisansakun et al. 2015) in its raceme inflorescence, shape of flower, and short fusiform capsule. The short fusiform capsule and the 4-colpate pollen grains of the new species support its placement in the subgenusImpatiens (Yu et al. 2015). In addition, *I.jenjittikuliae* has seeds coated with inflated cells with granulate walls similar to those described in species, such as *I.napoensis* Y. L. Chen, within the sect.Uniflorae (Janssens et al. 2012; Yu et al. 2015).

The cross sections of the stem of *I.jenjittikuliae* have shown that the new species is herbaceous, similar to the morphologically similar species in the sect.Uniflorae, *I.pulchra*, which also show in Lens et al. (2012) as *I.mengtszeana* Hook.f. (Lens et al. 2012; Ruchisansakun et al. 2015; Yu et al. 2015).

## Supplementary Material

XML Treatment for Impatiens jenjittikuliae
